# Plasmatic estradiol concentration in the mid-luteal phase is a good prognostic factor for clinical and ongoing pregnancies, during stimulated cycles of *in vitro* fertilization

**DOI:** 10.5935/1518-0557.20180005

**Published:** 2018

**Authors:** Rodopiano S. Florêncio, Melaynne S. B. Meira, Marcos V. da Cunha, Mylena N. C. R. Camarço, Eduardo C. Castro, Marta C. C. F. Finotti, Vinicius A. de Oliveira

**Affiliations:** 1 Humana Medicina Reprodutiva - Goiania, GO.; 2 Medicine School, Federal University of Goias.

**Keywords:** *In vitro* fertilization, estradiol in the luteal phase, mid-luteal phase, luteal phase support

## Abstract

**Objective:**

To evaluate the predictive efficiency of serum estradiol (E_2_) concentration in the mid-luteal phase regarding chemical, clinical, and ongoing pregnancies, in patients subjected to IVF/ICSI with fresh embryo transfer.

**Methods:**

One hundred and forty-three patients undergoing IVF/ICSI met all the inclusion criteria for the present study. Most of the patients used antagonists, final maturation was achieved with recombinant chorionic gonadotrophin (HCG), and embryo transfer took place on days 3 to 5, but mostly on day 4. The luteal phase was supplemented with estradiol valerate 6 mg/day and vaginal micronized progesterone 600 mg/day. There was no exclusion of patients in the embryo transfer group due to age or ovarian reserve. All patients with estradiol and chorionic gonadotrophin (βHCG) dosage on the day of transfer, day 7, were included. We assessed the following variables, initially regarding age: number of eggs collected, formed embryos, embryos transferred, day of transfer, transfer type, estradiol and chorionic gonadotropin. Next, we evaluated these elements at three different ranges of estradiol concentrations (<200 pg/ml, 200-500 pg/ml, and >500 pg/ml), comparing these parameters in pregnant (P) and non-pregnant (NP) patients.

**Results:**

Data analysis by age group in P and NP patients showed significant differences in the mean values of the variables E_2_ and βHCG, TD7. Mean serum estradiol levels in P and NP in the three age groups were: <35years, 835/417 *p*=0.0006, 35-39 years 833/434 *p*=0.0118, >39 years, 841/394 *p*=0.0012. There was also a significant difference in pregnancy rates in the group >500 pg/ml of estradiol concentration (63.4%, *p*=0.0096). The likelihood of chemical and clinical abortions for the estradiol ranges were: 38.46%, involving the two first ranges versus 15.15% for a concentration >500 pg/ml, *p*=0.0412 and 5.26% for a concentration >900 pg/ml, *p*=0.0105. The Pearson correlation coefficient for HCG and estradiol was r = 0.5108.

**Conclusion:**

This study showed the prognostic value of E_2_ in the mid-luteal phase (TD7) for chemical, clinical, and ongoing pregnancies, and its concentration suggested that there is a moderately positive correlation with βHCG levels.

## INTRODUCTION

Assisted reproduction treatments have achieved important positive results in recent years. Researches have evaluated several success factors that might interfere with the outcomes to help professionals achieve a better understanding of the whole process and improve it. Among those factors, serum estradiol concentration, both in the follicular phase (the initial phase or the day of chorionic gonadotrophin (HCG) administration for final maturation), and the mid-luteal phase have been investigated.

Some authors have investigated early follicular estradiol levels as a prognostic factor for pregnancy in cycles induced for in vitro fertilization, with or without intracytoplasmic sperm injection (IVF/ICSI). Moreover, such studies only used the agonist to block pituitary activity (Phelps *et al.*, 1998; Khalaf *et al.*, 2000). Studies with estradiol on the HCG day have not found any prognostic value (Erzincan *et al.*, 2014; Huang *et al.*, 2015). Another way of evaluating estradiol concentration (E_2_) as a prognostic tool is to calculate the rate of estradiol, comparing it on the day of HCG administration with the level obtained in the mid-luteal phase. Sharara *et al.* (2001) postulated that E_2_ ratios >5 could compromise endometrial quality. Hung Yu Ng *et al.* (2000) found no statistical difference in pregnancy rates with E_2_ ratios ≥5 or below this level. Several authors studied the variation and average serum E_2_ during luteal phases in natural cycles (Lenton *et al.*, 1982; Baird *et al.*, 1997) and induced cycles for IVF/ICSI after the pituitary activity was blocked (Hutchinson-Williams *et al.*, 1989; Balasch *et al.*, 1995, Aktan *et al.*, 2004; Friedler *et al.*, 2005; Ganesh *et al.*, 2008). These authors suggested that serum E_2_ levels in the luteal phase were higher when pregnancy occurred, as a reflection of trophoblastic gonadotropin (HCG) production in natural or induced cycles. In addition, Balasch *et al.* (1995) and Csemiczky *et al.* (1996) found a strong predictive value for clinical and ongoing pregnancies in relation to this hormone in the mid-luteal phase. 

Greb *et al.* (2004) demonstrated that E_2_ levels behaved distinctly when comparing conceptive and nonconceptive cycles on day 4 after embryo transfer (TD4), and that the mean value was higher until day 14. They reported that, in pregnant (P) cycles, although E_2_ levels start to increase on TD4, it was more evident on TD6; whereas in NP cycles its level was decreased. They also reported that in the luteal phase of cycles supplemented with HCG, E_2_ values were fixed as of TD6, and there were no cases of E_2_ alterations. Ganesh *et al.* (2008) compared the levels of E_2_ in P and non-pregnant (NP) patients on days 0, 7 and 14 in relation to TD after the IVF/ICSI procedure. They found similar E_2_ values on day 0 for both P and NP, and significantly higher levels for P on days 7 and 14. Hung Yu Ng *et al.* (2000) compared the mean level of E_2_ on TD6, and they reported that in cycles with HCG in the luteal phase, E_2_ concentrations were not significantly different in the two groups. Fatemi *et al.* (2007) reported that the addition of 4mg/day of E_2_ to progesterone in the luteal phase, produced higher levels of E_2_ on TD5. Despite these apparent evidences of the prognostic value of E_2_ in the luteal phase, the authors did not mention the benefits regarding the likelihood of pregnancy through the systematic use of E_2_ on different routes of administration (Zegers-Hochschild & Altieri, 1995; Fatemi *et al.*, 2006; Engmann *et al.*, 2008; Serna *et al.*, 2008, Gelbaya *et al.*, 2008). The estradiol concentration would only be a consequence of embryonic implantation, resulting in HCG production. Other authors (Gorkemli *et al.*, 2004; Lukaszuk *et al.*, 2005, Kutlusoy *et al.*, 2014) have found statistical significant differences in pregnancy likelihood with the addition of E_2_ or high doses of phytoestrogens to progesterone. Fujimoto *et al.* (2002) found a prognostic value of E_2_ levels above 500 pg/ml, with a significant higher pregnancy likelihood. In addition, they showed that E_2_ values below100 pg/ml during the mid-luteal phase meant lower pregnancy likelihood and this could be fixed in a later cycle using HCG associated with progesterone. DiLuigi *et al.* (2010) suggested that E_2_ concentrations in the luteal phase should be kept above 200 pg/ml in patients who used agonist for final maturation with estradiol and progesterone supplementation. 

These findings motivated us to evaluate our data retrospectively to determine whether E_2_ concentrations 7 days after embryo transfer (TD7) in P and NP patients within three age groups and three different E_2_ concentration ranges, from patients subjected to IVF/ICSI procedures, would be associated to chemical, clinical, and ongoing pregnancies.

## MATERIAL AND METHODS

One hundred and forty-three patients underwent ovulation induction by controlled ovarian hyperstimulation for IVF/ICSI from January 2010 to December 2012 due to artificial insemination failures, ovarian endometriosis and/or deep endometriosis, post-infection tubal factor infertility or salpingectomy, male factor infertility indicated by the ejaculate analysis, or post epididymitis or testicular biopsy.

All patients signed an informed consent form for anonymous retrospective data analysis.

Inclusion criteria: 1- Patients subjected to IVF/ICSI and transfer of fresh embryos aged between 23 and 45 years. Patients followed by the same examiner at all clinical stages, represented 20% of all procedures performed in the clinic during the study period. We included patients with low, normal, or high ovarian reserve who underwent routine hormonal dosages in the luteal phase.

Exclusion Criteria: 1- Egg recipients 2- Incomplete or missing medical exams.

In summary, IVF/ICSI cycles consisted of: priming with oral contraceptive pills in the pre-induction period for 12 to 21 days. We performed basal ultrasound scan on the last day of the pill, or at the beginning of the menstrual cycle. Ovulation induction was performed with recombinant or urinary gonadotropin in all patients in a 150 to 300 IU daily dose starting on the 2^nd^ day of the cycle. In the agonist group, we used 0.05 ml of subcutaneous leuprolide acetate (Lupron Kit^®^) daily, starting 4 days before the pill administration was interrupted. It was reduced to half of the initial dose after 7 days of treatment. In the antagonist group, we used subcutaneous administration of Cetrorelix (Cetrotide^®^) or Ganirelix (Orgalutran^®^), in a flexible regimen when follicles reached 12-14 mm of average diameter. When follicles reached a mean diameter of 19 to 20 mm, we administered recombinant chorionic gonadotrophin 250 mcg (Ovidrel^®^) or agonists (0.4 ml leuprolide acetate or 0.2 mg triptorelin) for those patients with ovarian hyperstimulation syndrome (OHSS) risk. The collection was performed 35-36 hours after HCG or leuprolide acetate injection, in most cases manually, and in a small number of cases with a medical suction pump. The eggs were injected 2 to 3 hours post collection or inseminated, in some cases of excellent semen quality. Fertilization was assessed after 19-22 hours. The embryos were transferred after 2 to 5 days, preferably 2 embryos, but 3, in certain special cases. Surplus embryos were frozen on days 3, 4, 5 or 6 post-collection. All patients undergoing embryo transfer used 2 mg of oral estradiol valerate and 200 mg of micronized vaginal progesterone every 8 hours or injectable 50 mg/day, in the second phase, starting on the collection day. We used transdermal estradiol (Estradot 100^®^), one adhesive daily, in the luteal phase, for patients who underwent agonist treatment for final maturation. We rarely used Ovidrel^®^ 50 mcg, on the day of ovum pick up, for patients at risk of OHSS who used agonist for maturation. Patients undergoing embryo transfer were submitted to estradiol, progesterone and chorionic gonadotropin (βHCG) dosing on day 7 post-transfer (TD7), and then progesterone and βHCG 14 days, after embryo transfer, to assess chemical pregnancy. We used βHCG >25 mUI/ml as chemical pregnancy criteria. When pregnancy was confirmed, we performed endovaginal ultrasound after 10 (clinical pregnancy) and 20 days (for heart beat) after the last βHCG dosing. We consider it to be an ongoing pregnancy, from 12 weeks on.

Estradiol concentration was measured in a Roche Modular Electrochemiluminescence device, the intra-individual variation was 18.1% and bias corresponded to 6.7%.

Retrospective analysis of the serum estradiol levels on TD7 +/- 1 (TD7) and other data that composed the variables were extracted from Excel spreadsheet.

**Figure 1:**
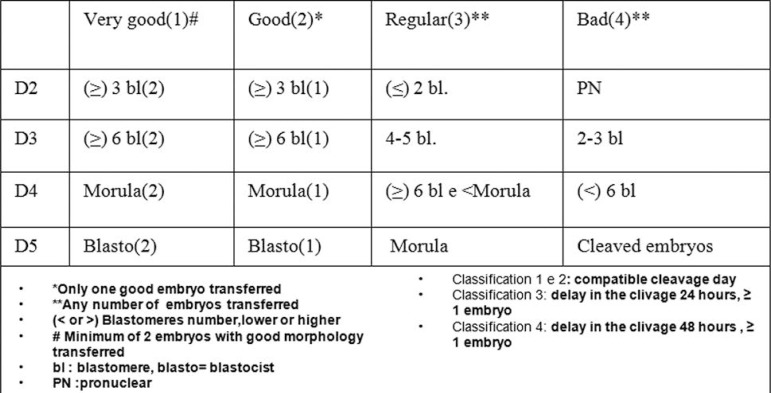
Embryo transfer classification, fresh (number of embryos, number of blastomeres, transfer day) Humana 2

We assessed the mean serum estradiol concentration, progesterone and quantitative βHCG on the day stipulated above, although we did not evaluate progesterone ratio correlation in this publication (Table 1). According to age range (<35 years, 35-39 years, >39 years) we evaluated the following variables: mature eggs (M2) injected, embryos obtained, embryos transferred, day of transfer, percentage of transfers type 1, 2,3,4 (our private clinic classification based on: the number of embryos transferred, number of blastomeres in each embryo considering the TD regardless of the degree of fragmentation - Figure 1). In addition, we evaluated the mean estradiol and chorionic gonadotropin levels in P and NP groups.

**Table 1:** ICSI. Variables associated with pregnancy likelihood in pregnant and non-pregnant women according to age groups (average).

Variables	<35 years (n=80)			35-39 years (n=42)			>39 years (n=21)		
	P	NP	*p*	P	NP	*p*	P	NP	*p*
Age	30.64±31.05	31.05±2.24	0.2565	37.43±1.49	36.88±1.36	0.1192	40.8±1.16	41.4±1.50	0.1213
Injected M2 oocytes *	10.73±5.34	9.44±6.78	0.1757	4.68±2.44	8.03±5.07	**0.0103**	5.00±1.09	4.50±2.57	0.3461
Embryos D3	8.11±4.73	6.24±5.76	0.0596	3.68±2.25	5.51±4.54	0.0772	3.41±1.01	3.25±1.82	0.4345
Transferred embryos	2.71±0.73	2.42±0.67	0.0353	2.25±0.75	2.34±0.78	0.3516	2.80±0.74	2.37±0.69	0.1398
Day of transfer	3.45±0.76	3.44±0.81	0.4889	3.37±0.85	3.42±0.74	0.4262	2.60±0.48	3.13±1.10	0.0989
Type of transfer	1.20±0.41	1.37±0.81	0.1207	1.46±0.49	1.57±0.68	0.2997	1.40±0.48	1.62± 0.92	0.3131
E_2_ (pg/ml) in MLP	835.26±724.93	417.81±222.90	**0.0006**	833.14±640.15	434.61±415.52	**0.0118**	841.04±351.15	394.40±189.60	**0.0012**
βHCG (mUI/ml) in MLP	20.83±21.82	0.70±1.59	**0.0001**	21.49±22.25	1.39±1.95	**0.002**	16.6±9.60	3.49±3.49	**0.0182**

* injected and rarely inseminated M2=mature E2=estradiol: MLP=mid-luteal phase (transfer day+7, TD7), *T-Test*=T-Test or Welch.

**Figure 2:**
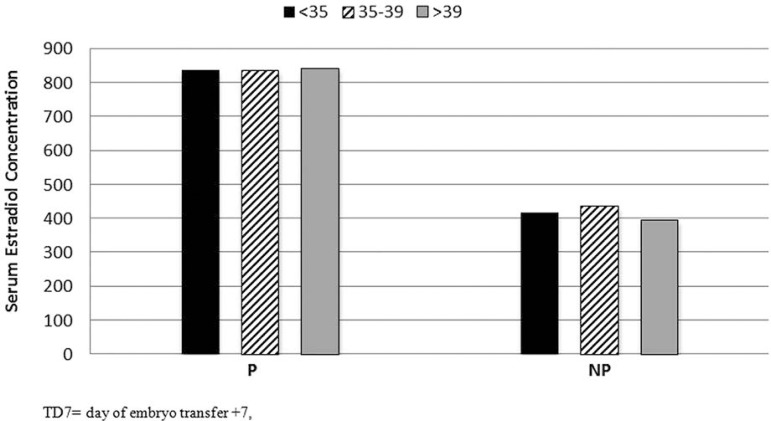
FIV/ICSI. Serum estradiol concentrations (picogram/ml) DT7, in pregnant women (P) and non-pregnant women (NP) according to three age ranges

**Figure 3:**
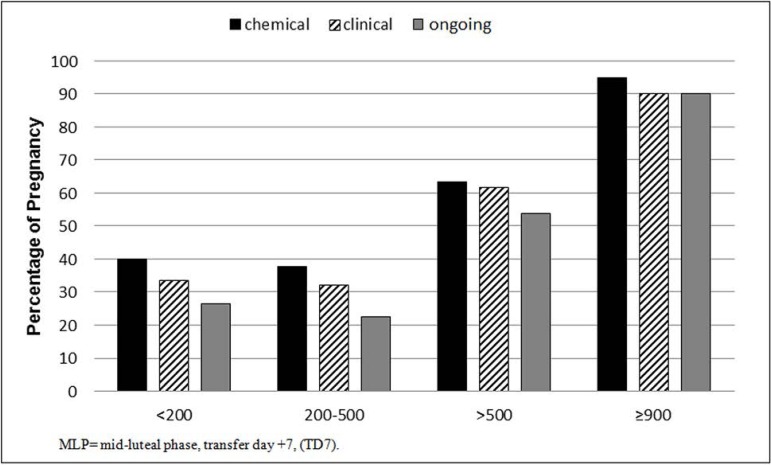
FIV/ICSI. Pregnancy rates in four different estradiol concentrations, picogram/ml, MLP, in patients ≤ 39 years

We evaluated the TD7 estradiol concentration at different ranges (<200, 200-500 and >500 pg/ml - Figure 2) and their relation to pregnancy prognosis. We also analyzed variables that could interfere with those concentrations and, in addition, we assessed P and NP by age group in patients up to 39 years of age. Figure 3 shows chemical, clinical, and ongoing pregnancy rates, in patients, according to estradiol concentrations. The addition of E_2_ concentrations, above 900 pg/ml, emphasizes the marker's prognostic value. 

The T-test was used to evaluate differences between the groups, the Welch's T-test was used for unequal sample sizes and Chi-square test with or without Yates correction and Fisher's exact test - to compare proportions. We applied Statistics for Excel and GraphPad software (QuickCalcs) to analyze the data. Significance for *p*<0.05.

## RESULTS

One hundred and forty-three patients included, according to the selection criteria, took part in this study. According to the age range there were: 80 patients <35 years, 42 between 35 and 39 and 21 >39 years of age. Data analysis of the age groups in P and NP (Table 1) showed significant differences between the variables E_2_ and βHCG on TD7. We found statistical differences in patients up to 39 years old related to the following variables (Table 2): a. among patients with E_2_ concentration <200 pg/ml, only βHCG (10.05/0.312; *p*=0.0247) between P and NP groups; b. in the group with E_2_ concentrations from 200 to 500 µg/ml, embryos formed (7.23/5.18; *p*=0.0143), embryos transferred (2.8/2.3; *p*=0.0134) and βHCG (10.59/0.79; *p*<0.0001) between P and NP groups; c. E2 concentration >500 µg/ml group, transfer type (1.18/1.47; *p*=0.0471), estradiol (1444/662.66; *p*=0.0042), βHCG (19.30/1.12; *p*<0.0001) between P and NP groups [Figure 2 shows a statistically significant difference in mean estradiol levels in P and NP (<35 years, 835/417; *p*=0.0006), (35-39 years 833/434; *p*=0.0118), (>39 years, 841/394; *p*=0.0012)]. Figure 3 shows chemical, clinical, and ongoing pregnancy rates within the three estradiol concentration ranges, and no difference between the groups <200 and 200-500 pg/ml (40%/37.7%), but significant difference for E_2_ concentrations >500 pg/ml (63.4%, *p*=0.0096), and a significant difference for the additional group ≥900 pg/ml (95%, *p*<0.0001). These results enabled us to calculate the likelihood of chemical and clinical abortions in the three concentration ranges (38.46% for the first 2 ranges versus 15.15% for concentrations >500 pg/ml, *p*=0.0412 and 5.26% for concentrations >900 pg/ml, *p*=0.0105. The Pearson correlation coefficient for HCG and estradiol was r=0.5108.

**Table 2:** ICSI. Variables associated with pregnancy likelihood according to estradiol concentration in pregnant and non-pregnant women <=39 years (average).

Variables	<200pg/ml			200-500pg/ml			>500pg/ml		
	P	NP	*p*	P	NP	*p*	P	NP	*p*
Age	30.66±5.18	32.77±3.61	0.2014	33.65±3.88	33.30±3.39	0.3697	32.27±3.49	33.68±3.49	0.1013
Collected oocytes	12.83±6.43	14.22±7.28	0.3649	15.75±12.45	10±6.90	**0.0195**	11.60±7.81	14.53±8.04	0.1069
Injected M2 oocytes*	7.57±4.13	10.00±7.26	0.2420	10.25±7.04	7.84±6.38	0.0891	8.57±4.48	9.57±5.15	0.2372
Embryos D3	4.50±2.21	7.22±6.92	0.2012	7.23±5.26	5.18±4.60	**0.0143**	6.57±3.87	6.94±5.47	0.3904
Transferred embryos	2.66±0.47	2.44±0.68	0.2651	2.80±0.74	2.30±0.75	**0.0134**	2.45±0.78	2.47±0.68	0.4654
Day of transfer	3.33±0.47	3.44±0.49	0.3467	3.35±0.72	3.24±0.77	0.4096	3.52±0.84	3.72±0.80	0.2199
Type of transfer**	1.66±0.74	1.33±0.47	0.1707	1.25±0.62	1.48±1.01	0.1827	1.18±0.45	1.47±0.75	**0.0471**
E2 (pg/ml) in MLP	132.42±52.23	105.11±53.55	0.1897	356.16±86.08	325.17±77.34	0.0968	1444.98±1185.60	662.66±119.04	**0.0042**
βHCG (mUI/ml) in MLP	10.05±8.44	0.312±0.40	**0.0247**	10.59±8.98	0.79±1.43	**0.0001**	19.30±11.54	1.12±2.09	**0.0001**

E_2_=estradiol, MLP=mid-luteal phase (transfer day+7, TD7), βHCG=chorionic gonadotropin,

* injected or inseminated,

** Human embryo transfer classification 2.

## DISCUSSION

Researchers in the field of assisted reproduction have been seeking to determine prognostic factors for success in IVF/ICSI for several years. Among these factors, serum estradiol concentrations in the follicular phase on the final maturation HCG administration day, and during the mid-luteal phase, has been extensively investigated, but mainly during the luteal phase, when the cycle can be evaluated, besides having the possibility of fixing this phase in the next cycle, if necessary.

The initial follicular phase was studied by Phelps *et al.* (1998) and Khalaf *et al.* (2000) who found a poor prognosis in pregnancy likelihood when estradiol levels on day 4 or 5 of the cycle were lower than 75 pg/ml in their first study and 50 pg/ml in the second; however, the studies were performed with agonists in a long-time frame protocol. On the day of final maturation of induced cycles (Erzincan *et al.*, 2014), before or after HCG administration, Huang *et al.* (2015), found no difference in pregnancy likelihood between the estradiol concentration groups <2000, 2000-4000 and >4000 pg/ml.

Several authors have reported higher levels of estradiol in the mid-luteal phase of conceptive cycles, both for natural (Baird *et al.*, 1997), or hyper stimulated ones, in patients undergoing IVF/ICSI, and having used HCG for final maturation (Balasch *et al.*, 1995; Greb *et al.*, 2004; Ganesh *et al.*, 2008; Moini *et al.*, 2011), without exogenous estradiol administration. Our results confirm those authors' findings, in a very clear and significant way, and it can be seen in Figure 2 (in correlation to age groups). The E_2_ concentration averages ranged from 831 to 841 pg/ml, in the P group, and 394 to 434 pg/ml in the NP group (*p*<0.001) (age groups). Beckers *et al.* (2003) carried out a prospective study regarding IVF/ICSI, and compared cycles with 150 IU/day of recombinant gonadotropin associated with antagonist, to block pituitary activity. Three groups were classified for the final maturation: 1-recombinant HCG 250 mcg (r-HCG), 2-recombinant LH 1 mg (r-LH) and 3-triptorelin 2 mg. No patient used drugs in the luteal phase. They evaluated the hormonal profile during the luteal phase and the duration of such phase. They reported that the luteal phase duration was longer with r-HCG (13 days), and the lowest duration was with triptorelin (9 days). E_2_ and progesterone profiles were reasonable with HCG and poor with r-LH and triptorelin. Pregnancy rates were extremely low. Their study showed the need for progesterone replacement in all IVF/ICSI cycles, in which pituitary activity was blocked. The use of E_2_ in daily doses of 4 to 6 mg/day to improve the luteal phase is a controversial topic. Fatemi *et al.* (2007), Ceyhan *et al.* (2008), Aghahosseini *et al.* (2011), Lin *et al.* (2013) and Engmann *et al.* (2008) in randomized studies, reported no benefit stemming from the administration of E_2_ in a dose of 4 mg/day. Other authors (Drakakis *et al.*, 2007; Jee *et al.*, 2010; Kwon *et al.*, 2013; Gizzo *et al.*, 2014; Zhang *et al.*, 2015) reported higher pregnancy likelihoods with the administration of E_2_, especially 6 mg/day of estradiol valerate. Higher pregnancy rates were also found in some studies involving estradiol patches, always associated with vaginal or injectable progesterone. There is controversy surrounding the use of E_2_ in the luteal phase when GnRH agonist is used for final maturation, but the administration of oral or transdermal estradiol hormones for those patients should not be questioned. Authors such as DiLuigi *et al.* (2010) recommended the maintenance of estradiol levels higher than 200 pg/ml in the luteal phase, associated with injectable progesterone. Our data confirms such authors' opinion. However, E_2_ levels at or below 500 pg/ml, showed no difference in the likelihood of pregnancy, even though our patients received aggressive E_2_ replacement in the luteal phase, associated with injectable progesterone, and apparently, a patient with100 pg/ml E_2_ on TD7 had the same likelihood of another patient with 500 pg/ml (Figure 3). We did not find any studies that investigated age group correlation to serum estradiol concentration. Our study showed no difference in estradiol concentration and age group, but there was a significant difference in those 3 age ranges between the P and NP groups (Table 1), showing that the production of estradiol was not altered by age, but only by the capacity of the lutein cells to respond to the production of trophoblast βHCG in a qualitative and quantitative way. Elements that may interfere with estradiol concentrations, apart from βHCG, have not been discussed in this paper but we plan to do it in another publication.

The variables analyzed in Tables 1 and 2 show data related to pregnancy likelihood. P and NP groups, according to the age group, showed that the analyzed variables, except βHCG and E_2_, did not present statistical difference, including mean age, and embryo transfer type. For example, the type of embryo transfer was numerically lower in the P group for the 3 age ranges (1.20/1.37, 1.46/1.57, 1.40/1.62), but such differences were not statistically significant. The remarkable statistical difference of E_2,_ followed by βHCG in the 3 age groups, suggests a positive correlation between the two hormones, although the correlation factor had presented a positive correlation of *p*=0.5108, a moderate one only.

Few authors, such as Fujimoto *et al.* (2002), studied estradiol concentration ranges to determine the chances of pregnancy in IVF/ICSI procedures related to the use of agonist in a long scheme. This group classified E_2_ concentrations into: <100, 100-500 and >500 pg/ml, on TD7, and they found pregnancy rates of 13.3%, 26.8% and 36.3%, respectively. In addition, they offered a second attempt of IVF in cases of failure for patients in the group with E_2_ <100 pg/ml. In this second cycle, they fixed the luteal phase with 3000 IU HCG on transfer days 1, 4 and 7. Such approach increased estradiol and progesterone levels, and pregnancy rates increased to 31.7%, against 13.7%, in the group that used only injectable progesterone.

We assessed the likelihoods of chemical, clinical, and ongoing pregnancies. We found similar pregnancy rates in the groups <200 and 200-500, but higher in the groups >500 pg/ml, *p*=0.0096 and ≥900 pg/ml, *p* <0.0001, in chemical, clinical and ongoing pregnancies (Figure 3), confirming the findings of Balasch *et al.* (1995). Another data for further investigation is the low abortion rate, in the group with E_2_ levels >500 pg/ml and >900 pg/ml, compared to the group of E_2_ ≤500 pg/ml (15.15% versus 38.46%, *p*=0.0412 and 5.26% versus 38.46%, *p*=0.0105).

## CONCLUSION

The present study shows the prognostic value of E_2_ in the mid-luteal phase, TD7, for chemical, clinical, and ongoing pregnancies. The E_2_ concentrations obtained, suggesting it had a positive correlation with βHCG levels.
